# Long-Term Effectiveness, under a Mountain Environment, of a Novel Conservation Nanomaterial Applied on Limestone from a Roman Archaeological Site

**DOI:** 10.3390/ma11050694

**Published:** 2018-04-28

**Authors:** Farid Elhaddad, Luis A. M. Carrascosa, Maria J. Mosquera

**Affiliations:** TEP-243 Nanomaterials Group, Departamento de Química-Física, Facultad de Ciencias, Campus Universitario Río San Pedro, Universidad de Cádiz, Puerto Real, 11510 Cádiz, Spain; farid.elhaddad@uca.es (F.E.); luis.martinez@uca.es (L.A.M.C.)

**Keywords:** archaeological site, hydrophobic/consolidant product, effectiveness, durability, in situ exposure

## Abstract

A novel alkoxysilane-based product was applied on limestone samples from a Roman archaeological site. The study consisted of an initial phase to evaluate site environmental conditions in order to choose the most suitable product type to be applied. The decay that was produced in the site is mainly caused by natural action, with water being the main vehicle for the decay agents. Thus, the effectiveness of an innovative product with hydrophobic/consolidant properties and two commercial products (consolidant and hydrophobic agent) were evaluated on limestone from Acinipo site, under laboratory conditions. Next, the long-term effectiveness of the three products under study was evaluated by the exposure of limestone samples in the archaeological site for a period of three years. Since the recognized incompatibility between alkoxysilanes and pure carbonate stones, the interaction between the products and the limestones was widely investigated. The results that were obtained allow for it to be concluded that the innovative product presents adequate compatibility and adherence to the limestone under study, producing a long-term effective, homogeneous, and continuous coating with a depth of penetration of up to 10 mm. However, the commercial products produced discontinuous aggregates on the limestone surface, did not penetrate into its porous structure and it did not produce long-lasting effects.

## 1. Introduction

Alkoxysilanes are commonly applied in the conservation of stone-based monuments. These products polymerize in situ within the pore structure of the disintegrating stone, and significantly increase the cohesion of the material [1]. Since water is the main vehicle of stone building decay agents, such as salts, microorganism, and other pollutants, organic components can be added to alkoxysilanes to produce hydrophobic products [2]. However, these products present two significant drawbacks: (1) their tendency to form brittle gels that are susceptible to cracking [3,4], and (2) their poor compatibility with carbonate stones [1,5].

Our research group has previously developed crack-free consolidants and hydrophobic products by mixing an aqueous surfactant solution and an alkoxysilane [6,7,8]. The surfactant, n-octylamine, prevents cracking of the gel because: (1) it increases the pore size of the gel network; and, (2) it decreases surface tension, both of which reduce capillary pressure, which is responsible for the cracking. Additionally, we observe a suitable effectiveness of these products in limestones [9]. Recently, other studies have used our strategy in order to obtain effective consolidant and hydrophobic products [10,11].

The effectiveness and the durability of novel conservation products are commonly evaluated under standard laboratory conditions [12,13,14,15,16,17]. However, their performance and durability are rarely tested in situ, under outdoor conditions [18,19,20,21,22,23,24,25]. Specifically, our research group has evaluated the performance of the novel consolidant consisting of a silica oligomer and a surfactant (n-octylamine). It was applied on two granitic monuments in Spain: a Romanesque church [18] and a medieval necropolis [19]. In the two cases, the novel product showed a better performance than that associated to the commercial products (an acrylic resin and an ethoxysilane) tested in the study.

Nwaubani et Dumbelton [20] evaluated the effectiveness of different commercial hydrophobic and consolidating treatments that were applied to the surfaces of three historic buildings and monuments in the UK. The surface treatments were applied in the range of 7–18 years at the time of testing. The results that were obtained highlighted that some treatments were deteriorated and a re-treatment should be applied.

Bonazza et al. [21] evaluated the efficiency and durability of two calcium-based consolidant on Carrara marble samples after outdoor exposure in four different European cities for 11 months. Both of the conservative products were found to remain mainly on surface and did not penetrate deeply in the substrate. It promoted a significant loss of the consolidating layers for the samples that were exposed in the sites characterized by heavy rain events.

De Ferri et al. [22] prepared two hydrophobic products by mixing hydrophobic silica nanoparticles with oligomeric ethoxysilanes, or alternatively, with epoxy resins. The obtained products were applied on three different building stones (marbles and sandstone). The treated samples were placed outdoors for 4 months, significantly reducing their hydrophobic performance.

Cappelletti et al. [23] also produced hydrophobic coatings by mixing an oligomeric polysiloxane and TiO_2_NPs. The obtained products were applied onto marble dolostone. The samples were placed at outdoor conditions for 7 months. The treatment clearly prevented the growing of salts.

Corcione et al. [24] prepared UV-cured nano-filled products by mixing trimethylolpropane trimethacrylate with a vinyl-terminated polydimethylsiloxane and an organically modified boehmite. The products were applied on two different calcarenite stones with high porosity. For comparison, two commercial products (alkylsilane-based product and acrylic resins) were also applied. The treated stones were placed outdoors at the region of Lecce (Italy) for 10 months. All of the treated stones maintained their hydrophobic properties, whereas they were significantly reduced in the case of the commercial products.

Finally, Gherardi et al. [25] dispersed TiO_2_NPs in different siloxanes and organosiloxanes. The obtained products were applied on the façade of the Cathedral of Monza (Italy), which is composed of two kinds of marbles. The treated façade was evaluated for 12 months showing good effectiveness and durability for most of the evaluated products. Nevertheless, the authors highlighted that 12 months is not enough for performing a real durability test, being necessary to carry out the evaluation for three or five years. These studies about on site monitoring of the performance of conservation products were, in all the cases, below 1 year. Moreover, most of them evaluated performance evolution with a single property.

Recently, our research group carried out an ambitious project to evaluate the durability of an innovative alkoxysilane-based product that was prepared in our laboratory [8] with hydrophobic and consolidant performance under real conditions, in two different Archaeological sites. The first study was carried out on the Baelo Claudia site built of sandstone and was subjected to a coastal environment [26]. In the present work, we evaluate the effectiveness and durability of the product previously described [8], on a limestone from Acinipo archaeological site, subjected to a mountain environment. As a prior phase to this study, we evaluated the environmental conditions and discussed the possible decay mechanism of the limestone from the archaeological site under study in order to select a suitable product for its correct preservation. For comparison, two commercial products were also evaluated. The effectiveness of these products was tested on limestone samples that were kept in situ, under the archaeological site conditions for a three-year period. Evaluation was carried out after each year of exposure. Since the recognized incompatibility between alkoxysilanes and carbonate stones [9,27], the interaction between the products under study and the limestones was widely investigated.

## 2. Materials and Methods

### 2.1. Evaluation of the Archaeological Site Environment and the Acinipo Limestone

**Figure 1:**
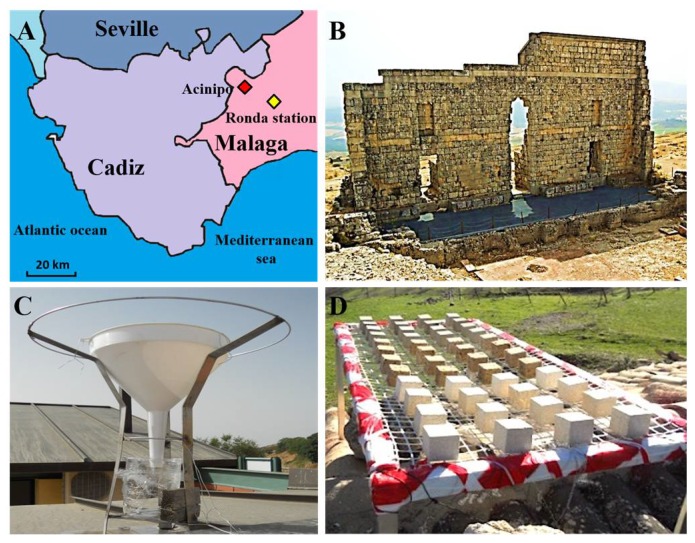
(**A**) Location of the archaeological site of Acinipo; (**B**) Roman theatre in the Acinipo site; (**C**) Particle collector; and, (**D**) The treated and untreated samples located at the archaeological site for three years.

The city of Acinipo was founded by the Romans in the first century BC. Nowadays, the Roman Theatre (Figure 1B) is the best-preserved building. In addition, ruins of the pre-Roman occupations, such as Neolithic Tartessian and Iberian villages (VIII-II centuries BC), are also preserved in this archaeological site. Acinipo is located in the south of Spain, specifically (Figure 1A) in the northeast of Ronda Mountains (Malaga) at 1000 m above sea level, and in an area of low pollution. Firstly, an analysis of the environment surrounding the site was carried out. Specifically, meteorological data of temperature, rainfall, and wind were collected for 24 months (corresponding to 2011 and 2012) from a National Institute of Meteorology station, which was situated near to the archaeological site (Figure 1A).

**Figure 2:**
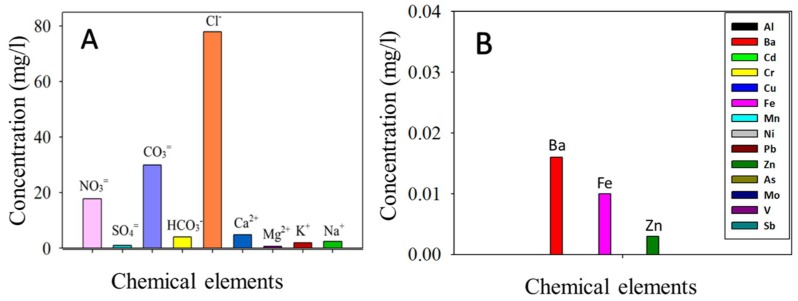
Chemical analysis of the soluble sedimentable particles (SP) fraction. (**A**) Ion concentrations; (**B**) Metal concentrations (The analysed metals are included in the inset).

In addition, an analysis of sedimentable particles (SP) that were collected during one month (August, 2012) at the archaeological site was carried out (see the SP collector in Figure 1C). Details about the SP analysis procedure are reported in a previous paper [28]. After collection, SP were rinsed with de-ionized water and filtered to separate the insoluble fraction. The chemical composition of the soluble SP fraction was determined, as follows: (1) Cl^−^ by titrimetry with silver nitrate; (2) CO_3_^=^ and HCO_3_^−^ by titrimetry with HCl; (3) SO_4_^=^ and NO_3_^=^ were quantified with a UV-Vis Spectrophotometer ZUZI 4210/50 ( I.C.T, S.L., La Rioja, Spain); (4) cations (Ca^2+^, Mg^2+^, Na^+^, and K^+^) by Atomic Absorption Spectroscopy (AAS) using a Perkin Elmer spectrophotometer (Perkin Elmer, Waltham, MA, USA); and, (5) identification of trace metals (see analysed metals in Figure 2) by Inductively Coupled Plasma-Mass Spectrometry (ICP-MS) with a Horiba Jobin Yvon Ultima 2 ICP (Horiba Scientific, Kyoto, Japan).

A JEOL 6460LV Scanning Electron Microscope (SEM, JEOL, Tokyo, Japan) was used to visualize the morphology of the insoluble SP fraction. Energy dispersive X-ray spectroscopy (EDX) spectra were recorded in order to determine their elemental composition.

### 2.2. Product Synthesis and Application on Acinipo Limestone Samples

A starting sol containing a commercial silica oligomer (Dynasylan 40, Evonik Industries, Essen, Germany) and a hydroxyl-terminated polydimethylsiloxane (PDMS from ABCR) in the presence of a surfactant (n-octylamine, from Aldrich) was synthesized, according to a previously described method [8]. According to their respective technical data sheets, Dynasylan 40 is a mixture of monomeric and oligomeric ethoxysilanes with a SiO_2_ content of 40%, and PDMS is an oligomer with a polymerization degree of 12 and an OH percentage ranging from 4 to 6% *w*/*w*. The synthesis route was as follows: A 1.57 M aqueous solution of n-octylamine was prepared. Next, PDMS, Dynasylan 40, and the aqueous n-octylamine solution were mixed and homogenized by high-power ultrasonic agitation (60 W·cm^−3^) for 10 min. PDMS and aqueous n-octylamine solution proportions were 10 and 0.075% *v*/*v*, respectively. The product was named UCA (after University of Cadiz).

The limestone was extracted from Acinipo theatre area and it was cut as 4 cm-cubed samples. Petrographic and mineralogical analyses were previously carried out on thin sections of stone using a Transmitted Light Microscope (Olympus BH-2). The UCA product was sprayed on the 4 cm-cubes using a pressure of 1.5 × 10^5^ Pa for 25 s. For comparison purposes, two popular commercial products, which were manufactured by Wacker (Munich, Germany) were also applied under the same conditions. Specifically, a consolidant product BSOH100 and a hydrophobic product BS290 were selected. BSOH100 is a solvent-free product, consisting of partially pre-polymerized tetraethylorthosilicate (TEOS) and dibutyltin dilaurate (DBTL) catalyst. BS290 is a solvent-free silane/siloxane mix. It was diluted in ethanol (12% *w*/*w*), following the recommendations of the manufacturer.

### 2.3. Assessment of Effectiveness and Durability

The treated samples were dried under laboratory conditions (20 °C, 60% RH) until constant weight was reached. This occurred one month after application. Effectiveness of the products applied on the Acinipo limestone was evaluated in the laboratory using three samples for each treatment. In addition, the durability of the products was evaluated in samples exposed to the environmental conditions of Acinipo archaeologic site. The study was carried out every year on a limestone sample, for a total period of three years. The following assessments were carried out on the treated limestone samples and their untreated counterparts. In the case of the samples that were exposed to archaeological site conditions, in the tests requiring the whole sample: evolution of mass, water absorption by capillarity, and water vapor diffusivity studies, one specimen per year was tested. In the other cases, the number of the replicates is described for each specific test.

#### 2.3.1. Limestone-Product Interaction

Uptake of products and their corresponding dry matter were determined. Both parameters were measured by the change in the mass of the specimens.

Mercury accessible porosity and porosimetric distribution were obtained by Mercury Intrusion Porosimetry (MIP). The test was carried out on three specimens per stone sample under study, each with a volume of around 1 cm^3^, using a PoreMaster-60 device from Quantachrome Instruments (Boynton Beach, FL, USA) comprising two measurement units: a low-pressure unit (Pascal 140), whose pressure range is between 0.69 and 350 kPa, and a high- pressure unit (Pascal 440), whose pressure range is between 0.1 to 420 MPa.

A JEOL Quanta 200 Scanning Electron Microscope (SEM, JEOL, Tokyo, Japan) was used to visualize changes in the morphology of the limestone samples. The samples were covered by a 12-nm layer of gold in order to improve their conductivity and prevent charge effects. Energy Dispersive X-ray Spectroscopy (EDX) spectra were recorded, on the whole SEM area under study, in order to elucidate the variations in surface elemental composition after the application of the products.

The topography of the stone surfaces was observed using Atomic Force Microscopy (AFM, Nanotec Electrónica S.L, Madrid, Spain) operated in tapping mode. The root-mean-square (RMS) roughness values were calculated from 2.5 μm × 2.5 μm images. Five scans were obtained for surface sample under study.

Fourier Transformer Infrared Spectra (FTIR) was employed to study the interaction between Acinipo limestone and the products under study using a FTIR-8400S (Shimadzu, Kyoto, Japan), 4 cm^−1^ in resolution, in the region from 4000 to 650 cm^−1^. 20 scans were performed per sample. The experiments were carried out in Attenuated Total Reflection mode (ATR). In order to enhance the intensity of the signals, the samples for FTIR were prepared. Specifically, the untreated stone sample powder was mixed with the products under study (as sols) at 50% *w*/*w* powder/product. In the case of BS290, it was mixed without previous dilution in ethanol. Next, they were left to gel and dry under laboratory conditions. Then, the obtained composites stone-product were powdered and analyzed by FTIR. In the case of durability studies, powder that was obtained from the surface of the samples at laboratory conditions and subjected to weathering at the archaeological site was employed. The powder was obtained by scratching the treated surface of the samples under study.

#### 2.3.2. Effectiveness of the Products on the Limestone

Improvement in mechanical properties of the treated stones and their untreated counterparts were evaluated by using the Vickers Hardness Test (Centaur RB2/200 DA, Metrol Centaur S.L., Vizcaya, Spain). Five indentations per each sample surface sample under study were carried out.

The adherence of the coatings to the stone surface was evaluated by performing a Peeling Test using Scotch Magic tape (3M). The test was carried out according to a previously reported method [29,30]. The attach/detach cycles were carried out on five different areas for each sample under study.

The effectiveness of the products in providing hydrophobic protection was characterized by the contact angle test, according to the sessile drop method, using a commercial video-based, software-controlled contact angle analyser, model OCA 15 plus, from DataPhysics Instruments (Filderstadt, Germany). The static contact angle values were determined on the stone surface. The advancing and receding contact angles were measured using the ARCA method included in the equipment software (OCA15 plus, DataPhysics Instruments, Filderstadt, Germany). The measurement was carried out on five points for each surface (the four corners and the centre).

To confirm the hydrophobic behaviour of the materials, stone samples were subjected to a test to determine the Capillary Water Absorption Coefficient (WAC), as recommended in UNE-EN [31] and the total water uptake was determined.

#### 2.3.3. Negative Effects Induced by the Products on the Limestone

The changes in color were evaluated by using a solid reflection spectrophotometer (HunterLab, Reston, VA, USA), ColorFlex model. The conditions used were as follows: illuminant C and observer 10°. CIE L*a*b* color space was used and colour variations were evaluated using the total color difference parameter (ΔE*) [32]. The measurement was carried out on five different points for each surface sample (the four corners and the center).

Water vapor diffusivity was determined using an automatic setup developed in our laboratory [33], based on the standard cup test, in 4 × 4 × 1 cm stone slabs.

#### 2.3.4. Evaluation of the Depth of Product Penetration

The penetration depth of the products was evaluated on the cross-sectional surfaces of the limestones by the static contact angle test carried out at different depths.

In addition, to confirm the penetration depth of the products under study, images of the cross-sections of the treated limestone samples and their untreated counterparts were obtained using a Nikon model SMZ800 stereoscopic microscope (Nikon, Tokyo, Japan), according to the following procedure: the samples were immersed in water. Then, they were cut and visualized by microscopy. The non-wetted area can be associated with the penetration depth of the products across the pore structure of the limestone.

## 3. Results and Discussion

### 3.1. Evaluation of the Archaeological Site Environment and the Acinipo Limestone

The meteorological data obtained during the sampling period (Figure S1 in supplementary materials) show the monthly maximum average temperatures ranging from 33 °C in August to 11 °C in February. The monthly minimum temperatures ranged from 17 °C in August to 0.5 °C in February. The distribution of the rainfall values during the period of study was completely heterogeneous. The period from June to August exhibited values near to 0 mm, whereas the highest rainfall was recorded in the months of April and October, with values of 90 and 80 mm, respectively. Concerning wind, it had a moderate speed (around 20 km/h) with North prevailing components.

The chemical analysis of the soluble fraction of SP (Figure 2A) revealed high concentrations of Cl^−^ (78 mg/L), CO_3_^=^ (30 mg/L), and NO_3_^=^ (18 mg/L), and concentrations of <5 mg/L of HCO_3_^−^, Mg^2+^, K^+^, Ca^2+^, Na^+^, and SO_4_^=^. The highest concentration of Cl^−^ can be related to the deposition of marine aerosol (NaCl) transported by wind (the distance to the sea is 40 km). The values that were obtained for the metals analysis (Figure 2B) show a low concentration of Ba, Fe, and Zn. According to Prieto-Taboada et al. [34], the absence of the following metal elements: Pb, As, Cu, Cr, Ni, Cd, Mn, and Sr, and the presence of a low concentration of Ba and Zn, all of them related to traffic pollution and nearby industrial activity, clearly highlights the absence of anthropogenic activities in the Acinipo site surroundings. In the case of the Fe, this is an abundant element on Earth, being part of many minerals. Thus, its presence cannot also be associated to anthropogenic activity.

**Figure 3:**
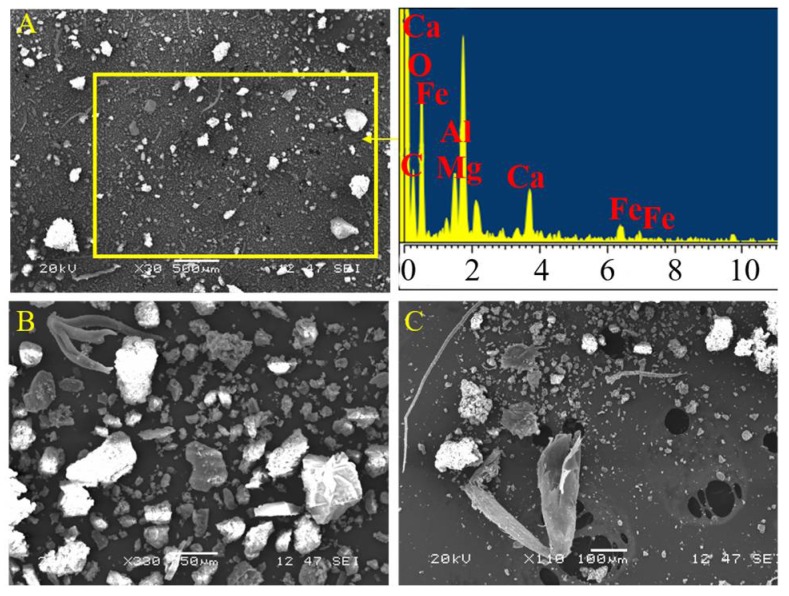
(**A**) Scanning electron microscope (SEM) images of particles obtained from the insoluble SP fraction and their corresponding energy dispersive X-ray spectroscopy (EDX) spectrum; SEM images of biological remains that were obtained from their insoluble SP fraction: (**B**) plant debris; and (**C**) insect remains.

The insoluble SP fraction images that were obtained by SEM are shown in Figure 3. Most particles observed are irregular with sizes below 50 µm and some larger particles with 200 µm in size (Figure 3A). The EDX analyses of this area show typical elements of natural environments (O, C, Si, Al, Ca, Mg, and Fe) [35]. Particles of biological origin, which are composed of insect remains, diatomite and plant debris, are also observed in the SP fraction (see Figure 3B,C).

The results previously described highlight that the Acinipo environment, in terms of climatic features, does not present extreme temperatures, and the rainfall and wind phenomena are moderate. In addition, the SP fraction analysis allows the conclusion that the Acinipo archaeological site is a natural environment without anthropogenic activity. Therefore, the main mechanisms of the Acinipo limestone decay could be associated with the crystallization of salts (mainly NaCl from marine aerosol) and the growth of microorganisms that are favored by the absence of extreme temperatures [36]. Following these conclusions, consolidant products with hydrophobic properties preventing water ingress into the stone pores, which is the vehicle of salts and microorganisms, were considered for Acinipo limestone conservation.

**Figure 4:**
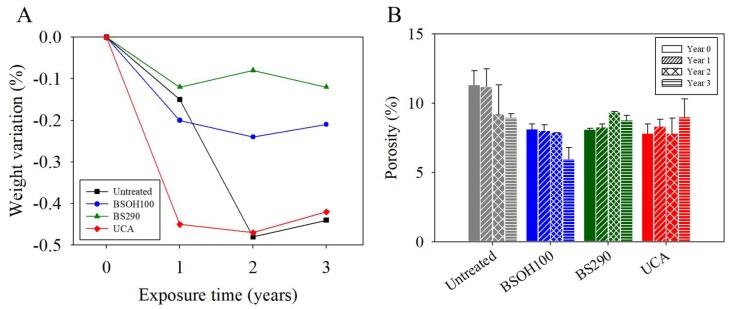
(**A**) Weight variation and (**B**) Porosity of the treated limestone samples and their untreated counterpart under laboratory conditions (0 year) and after each year of exposure to the environmental conditions of the Acinipo archaeological site.

Regarding the Acinipo limestone evaluation, the optical micrograph (Figure S2A) presents a bioclastic packstone that is characterized by an abundance of fragments of bryozoan-coralline algae up to 1 mm in length. In addition, echinoderms, foraminifers, and shell fragments are observed. The matrix of this stone is micrite, with part of this micrite being recrystallized to sparite or microsparite. The EDX spectra (Figure S2B) show that Acinipo Limestone is composed of calcite (CaCO_3_) and a very low proportion of quartz (SiO_2_). Finally, the limestone shows a medium porosity (11%, see Figure 4).

### 3.2. Assessment of Effectiveness and Durability

#### 3.2.1. Limestone-Product Interaction

The Table S1, which is included in supplementary information, shows the uptake (U) and dry-matter (D) values for the products under study. The uptake values of UCA and BSOH100 were similar due to their close viscosity values [26], whereas BS290 was slightly lower due to the quick evaporation of the ethanol solvent. However, significant differences were observed for the dry-matter values. Specifically, BS290 showed the lowest dry-matter value as a consequence of its dilution in ethanol [37]. Its D/U ratio (16%) demonstrated that practically all of the solvent was evaporated. In the case of the two solvent-free products (BSOH100 and UCA), they showed higher D/U ratios (45 and 69%, respectively). In this case, the mass reduction is due to the removal of water and ethanol, which is produced during hydrolysis and condensation reactions.

Figure 4A shows the evolution of weight for the samples under study with respect to their corresponding weight at year 0. A scarce weight loss (<0.5% *w*/*w*) was observed for all of the samples under study. By comparing between samples, the highest reduction corresponded to the untreated sample and its UCA treated counterpart. In the case of the UCA product, this reduction took place in the first year of exposure to the archaeological site. This behavior can be associated with the condensation/drying processes of the products being complete during the first year. In a previous paper [8], the presence of ethoxy groups from non-hydrolyzed oligomers was observed in the UCA product after six months of the synthesis, confirming the incomplete condensation of the product. In addition, a significant reduction in weight of the sample treated with the UCA product was observed after the first year of exposure at Baelo Claudia archaeological site due to the condensation/drying process [26].

Finally, the samples that were treated with BS290 showed a similar trend to their untreated counterparts, which can be related to the formation of a thin and discontinuous coating of the product being removed during the in situ exposure. During the third year, an increase in weight was observed as a consequence of the deposition of atmospheric particles and biological materials [34]. This result confirms that BS290 does not produce an effective and long-lasting treatment for the limestone under study.

**Figure 5:**
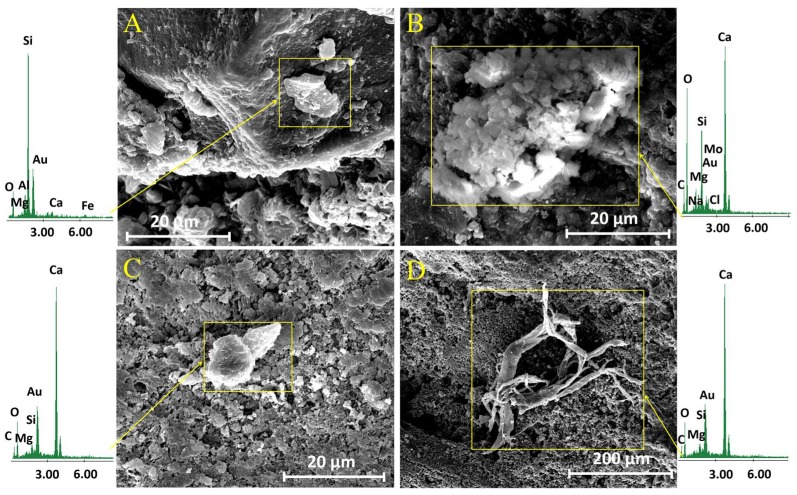
SEM images of the mineral particles (**A**–**C**) and (**D**) particle of biological remains on the surface of the untreated limestone, and their corresponding EDX spectra, after three years of exposure to the environmental conditions of the Acinipo archaeological site.

In order to confirm the deposition of particles, after three years exposure in situ, the surfaces of the untreated samples were visualized by SEM. Figure 5 shows the obtained micrographs and their corresponding EDX analysis. These images (Figure 5A,B) clearly show the presence of particles with a diameter of around 10–20 μm, deposited on the limestone surface. The analysis of these particles by EDX shows the presence of Si, Al, Ca, Mg, and Fe, elements typical of natural environments without contamination [35].

In addition, the presence of NaCl (halite) (Figure 5C) was observed on the limestone surface. EDX analysis confirms its presence at low concentration. Na^+^ and Cl^−^ were also observed in the environmental study of Acinipo. The low NaCl concentration in the limestone can be related to its high solubility, which favors its dissolution and elimination by rain [38]. The presence of biological particles (Figure 5D) was also observed by SEM. Similarly, atmospheric particles, halite, and biological material were observed in the surface of the limestones treated with BS290 after three years exposure.

Micrographs of untreated limestone surface and its treated counterparts, after three years exposure, are shown in the Figure S3. The growth of microorganisms is clearly observed in the untreated and the commercial products treated samples, whereas it is not observed in the sample that was treated with UCA product. These results confirm that UCA produces an effective coating on the surface, which prevents water presence and the subsequent growth of microorganism for at least 3 years. To confirm the presence of these microorganisms, the colonized surface of the untreated limestone was put in contact with a favorable culture medium (potato dextrose agar, PDA) for one week. A significant fungal hyphal growth can be observed in the culture medium, confirming that live microorganisms are present in the limestone (Figure S4 in supplementary information).

To evaluate the changes in the limestone porous structure during exposure to the environmental conditions, a Mercury Intrusion Porosimetry (MIP) test was carried out on samples that were extracted from the limestone surface. The changes generated in the MIP porosity can be explained by the presence of the product in the pores on the surface region of the limestone [39], which modifies its total porosity and pore diameter [40]. The average porosity values are presented in Figure 4B. After the treatments, all of the products reduced the total porosity by approximately 30%, with no significant differences between the products.

During three years of exposure to environmental conditions, the untreated samples and those that were treated with BSOH100 showed a gradual decrease in their total porosity values (Figure 4B), with slight fluctuations, as a consequence of deposition of salts and atmospheric particles in the pores of the stone [41], and as previously observed by SEM (Figure 5). In addition, the presence of biological material can also reduce the porosity and the pore size distribution of the stone [42].

Finally, the stone that was treated with the hydrophobic products BS290 and UCA do not present a reduction of porosity with exposure time, and their value remains practically unchanged, due to the hydrophobic effect of these coatings preventing the penetration of aqueous solutions containing salts or other contaminants into the stone. Striegela et al. [43] highlights that the ingress rate of pollutants into the pore structure is significantly lower in the case of the stones treated with hydrophobic products.

Figure S5 in Supplementary Materials shows the distribution of the pore diameter obtained by MIP. The untreated sample, before in situ exposure, presented pores that were mainly located in two ranges: 0.03–0.5 and 0.9–5 μm, corresponding to 40% and 36% of the porous volume, respectively. In addition, 4% of the porosity is located in the microporous region (<0.01 μm) and 11% in the macroporous region (5–200 μm). In the case of the treated samples, in all cases, a significant reduction in the pores that were located between 0.9–5 μm is promoted, while an increase of porosity in the range 0.03–0.5 μm is observed, as a consequence of the partial pore block by the applied products [39]. The reduction is higher for the samples treated with the UCA product, and lower in the case of BS290. Regarding the reduction of porosity caused by the three products in the micropore fraction, significant differences were not observed. Finally, it must be mentioned that the macropore fraction was not modified by any of the applied products.

After archaeological site exposure, in all the cases, and especially in the untreated limestone and its counterpart that was treated with BSOH100, a significant decrease in the 0.9–5 μm pore size range to values in the 0.03–0.5 μm range was observed as a consequence of the deposition of salts and other previously discussed contaminants. For BS290 and UCA products, the changes that were observed were significantly lower. This confirms that material ingress into the pore structure is lower in limestone treated with these products.

**Figure 6:**
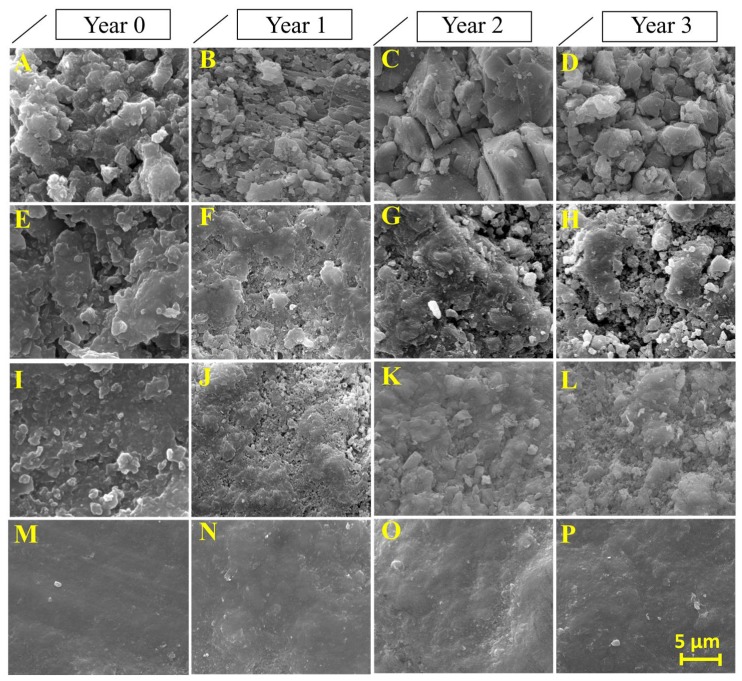
SEM images of the stone surface, before and after three years of exposure to the environmental conditions of the Acinipo archaeological site: (**A**–**D**) untreated; (**E**–**H**) BSOH100; (**I**–**L**) BS290; and, (**M**–**P**) University of Cadiz (UCA).

**Figure 7:**
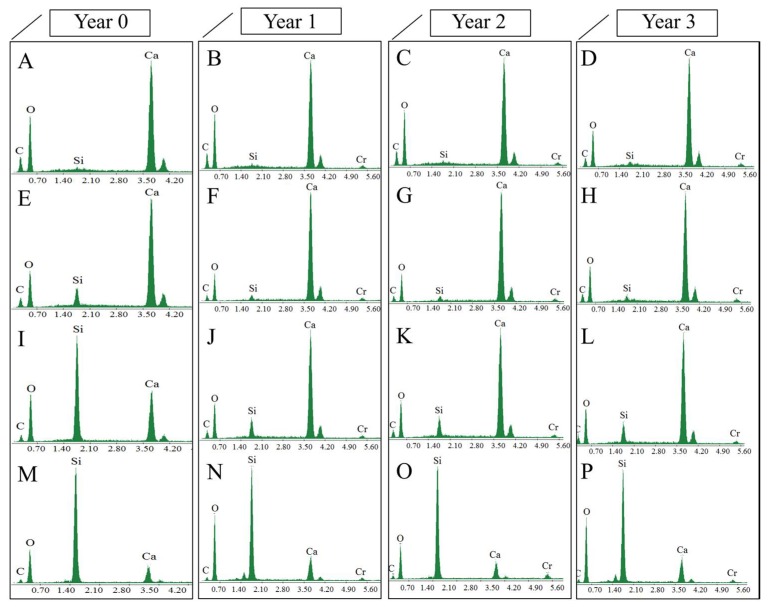
EDX spectrum of the stone surface, before and after three years of exposure to the environmental conditions of the Acinipo archaeological site: (**A**–**D**) untreated; (**E**–**H**) BSOH100; (**I**–**L**) BS290; and, (**M**–**P**) UCA.

The morphology and structure of the surfaces of the treated and untreated limestone samples were visualized by SEM, and their corresponding EDX spectra were obtained. The presence of the alkoxysilane-based products was evaluated from the Ca/Si ratio. The images obtained, and their spectra, are shown in Figure 6 and Figure 7. Under laboratory conditions, the limestone minerals are clearly observed in the untreated sample (Figure 6A). The three studied products significantly modify the surface morphologies of the treated samples. Specifically, the surface that was treated with BSOH100 (Figure 6E) shows a discontinuous coating, which is formed by isolated aggregates of the gel, with large areas of uncoated surface. The EDX spectrum (Figure 7E) presents a similar spectrum to that corresponding to the untreated sample, with a slight increase in Si peak intensity. It confirms the presence of an uncoated part in the analyzed sample. The surface treated with BS290 produced a coating of siloxane aggregates but it was not homogenous and continuous (Figure 6I). The EDX spectrum shows a clear Si peak associated with the siloxane aggregates (Figure 7I). The presence of Ca in the analyzed surface corroborates the existence of some discontinuity in the coating. The formation of discontinuous coatings from the two commercial products is due to their poor interaction with the limestone under study. The UCA product creates a continuous, dense, and homogeneous coating on the limestone sample (Figure 6M). The significant reduction in the Ca peak (Figure 7M) corroborates the presence of a homogeneous coating. The formation of a discontinuous coating in BS290 has been observed in a previous work [14] and is associated with the high dilution of the product in ethanol. The low dry matter of the coating confirms this trend.

In the case of the two commercial products, the formation of aggregates of isolated gel could also be related to the low compatibility between limestone and alkoxysilanes [1,5], which could prevent the formation of a continuous and well-adhered coating on the limestone. In a previous study, Zendri et al. [44] evaluated the reactivity between alkoxysilanes and calcium carbonate by using NMR techniques. The results showed that the presence of CO_3_^=^ modifies the reactivity of the silica precursor, promoting the reaction of short chains of the gel in order to generate isolated aggregates of product with low adherence to the substrate, as observed by SEM in this study (Figure 6).

After in situ exposure of the samples, the surfaces that were treated with BSOH100 and BS290 products clearly show a significant loss of the coating over exposure time, especially in the second and third years, where the presence of the mineral grains of the stone can be clearly appreciated. The loss of the coating is confirmed by the EDX spectra, where the Si peak was significantly reduced and the Ca peak was increased from one year of exposure. The surfaces treated with the UCA product do not show significant changes after three years of exposure, maintaining the intensity of the Si and Ca peaks without modification. It confirms that the UCA product is able to adhere firmly to the stone surface, providing resistance to alteration for a period over three years.

**Figure 8:**
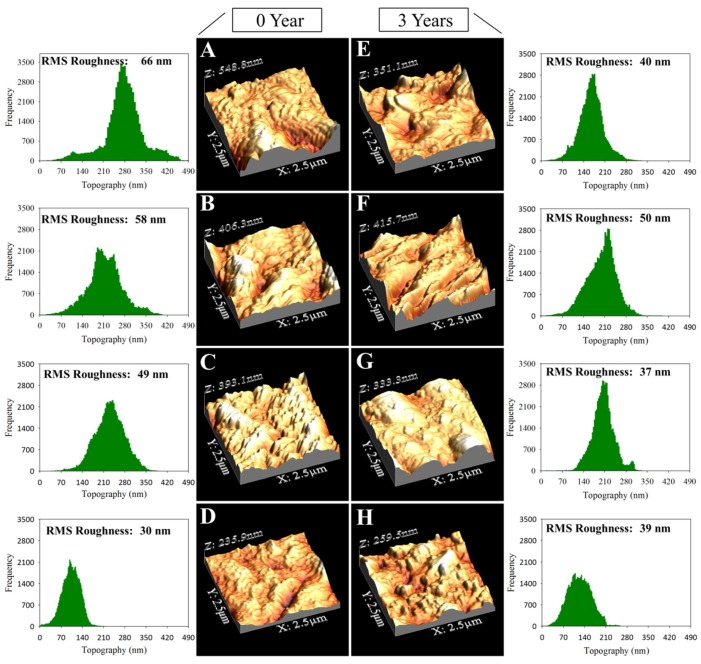
AFM (Atomic Force Microscopy) three-dimensional (3D) topographic images and their corresponding size distribution of the roughness on the limestone surfaces under study, before and after three years of exposure to archaeological site conditions: (**A**,**E**) untreated; (**B**,**F**) BSOH100; (**C**,**G**) BS290; (**D**,**H**); and, UCA.

In addition, the analysis of SEM images reveals a significant change in limestone roughness after exposure to the archaeological site conditions. For this reason, the surface roughness of the samples before and after their exposure for three years was studied. Figure 8 shows the three-dimensional (3D) images of the limestone surfaces obtained by AFM (Atomic Force Microscopy). The untreated stone surface presents a random roughness associated with the heterogeneity of the stone. The morphology of the limestone is slightly modified after the treatment with the commercial product. Figure 8 gives the size distribution for the roughness of the limestone surfaces under study, and the RMS roughness that was obtained. All of the applied products reduce the surface roughness of the limestone, creating coatings that tend to fill the existing holes in the untreated stone. The higher reduction in the roughness of the untreated stone was observed in the case of the samples that were treated with the UCA product (55% reduction). This reduction confirms the formation of a continuous and homogeneous coating, as observed by SEM (see Figure 6).

After three years of exposure to environmental conditions, the untreated samples showed a clear reduction in surface roughness. This can be associated with stone erosion, by the action of rain and wind, causing the loss of grains that were less attached to the surface or by mineral dissolution. In addition, this reduction of roughness could be associated with the deposition of particles and salts filling the spaces of the stone surface. The treated samples do not show a significant change in the roughness after three exposure years.

**Figure 9:**
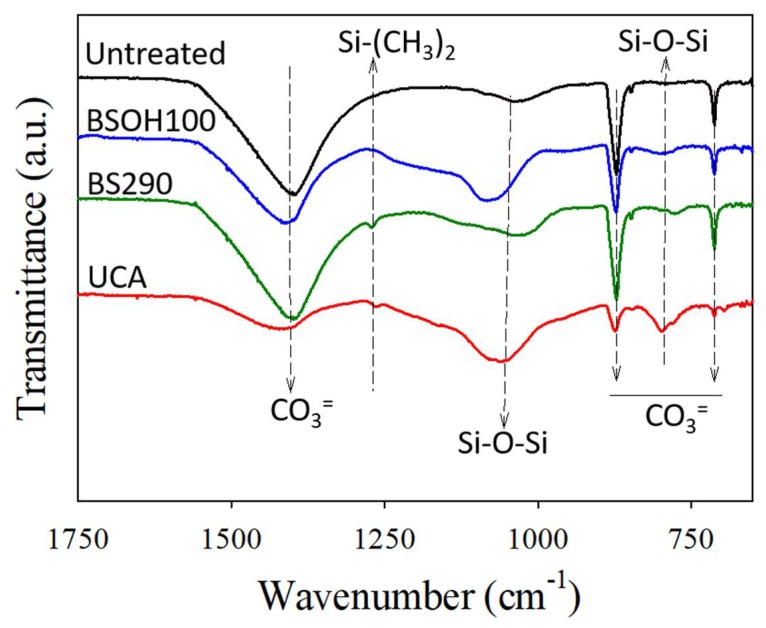
Fourier Transformer Infrared Spectra (FTIR) spectra of the treated and untreated limestones.

To confirm the compatibility between the carbonated substrate and the applied products, FTIR spectra were obtained (see Figure 9). In addition, Table S2 in supplementary information shows the relative intensity of the main siloxane band with respect to the main carbonate band for each spectrum. They confirm the composition of Acinipo limestone that was previously obtained by petrography (Figure S2). Specifically, the spectra of the untreated samples show bands at 712, 870, and 1400 cm^−1^, assigned to CO_3_^=^ [45,46,47]. It confirms that calcite (CaCO_3_) is the main component in the Acinipo limestone. In addition, a weak band was observed at 1080 cm^−1^, which is assigned to a Si′O′Si bond [7], and confirms the presence of a small proportion of quartz (SiO_2_) in the limestone.

In the spectra corresponding to samples of limestone mixed with the products, new bands were observed: a typical silica band, absent in the untreated sample, located at 800 cm^−1^, assigned to the bonding vibration of Si–O–Si [6,48]. This band confirms the presence of siloxane bonds from the products that are in the limestone. In a comparison between products, the intensity of this band was significantly higher in the spectrum of the sample containing the UCA product than those corresponding to the commercial products. In the case of the spectra corresponding to samples treated with the products BS290 and UCA, an additional band, which is located at 1265 cm^−1^, is attributed to Si–(CH_3_)_2_ bonds of the organic component that is present in both products and responsible for its hydrophobic properties [48,49]. In the UCA product, the presence of this bond confirms the integration of the PDMS into the silica gel, due to the existence of a co-polymerization process between the PDMS and the silicon oligomer [14,37].

On the other hand, a significant change in the intensity and position of the bands of CO_3_^=^ (located at 712, 870, and 1400 cm^−1^) with respect to those that were observed in the untreated and commercial products spectra is observed in the UCA spectrum. According to Wheeler et al. [50], the intensity of the bands of CO_3_^=^ that is present in calcite can be modified as a consequence of their interaction with another chemical species that produces a change in the counter ion bound to CO_3_^=^. In this previous work, the –NH_2_^+^ group of the aminopropyltriethoxysilane, used as the coupling agent, interacts with the CO_3_^=^ producing this effect. In the present study, the amino group of n-octylamine, which is present in the UCA product, could interact with the ion CO_3_^=^ and be responsible for the modification of the intensity of its peaks. Similar results were also observed by Xu et al. [51], as calcite interacts with an aminosiloxane.

In order to demonstrate the possible interaction between n-octylamine and Acinipo limestone, the stone powder was mixed with this catalyst in a limestone:n-octylamine ratio, 1:0.5. The Figure S6 in supplementary information shows the spectrum that was obtained after 24 h of interaction between the two species. For comparative purposes, the spectrum corresponding to the untreated limestone is also included.

The two spectra show the peaks corresponding to the limestone. In particular, the three bands characteristic of CO_3_^=^ and a weak band associated with Si–O–Si bonds [45,46,47], which are associated with the small proportion of quartz present in the stone [7]. In addition, the limestone-n-octylamine spectrum shows new bands that may be associated with the existence of interactions between these two species [52]. Specifically, the peaks that were observed at 2922 and 2852 cm^−1^ correspond to the asymmetric vibration of the CH_2_ and symmetric vibration of CH_3_ and CH_2_. These bands may be associated with the alkyl chain of the n-octylamine. On the other hand, the band observed at 3322 cm^−1^ can be associated with the N–H bond present in amides, and the bands at 1640 and 1563 cm^−1^ correspond to the vibrations of C=O and N–H, respectively, of secondary amides [53]. According to Demjén et al. [52], the formation of amides could be the result of the reaction of the CO_3_^=^ of the limestone with the amino group of n-octylamine. In addition, another band is observed at 1790 cm^−1^, which is associated with the symmetric vibration of imides, this band may be related to the possible interaction of the previously formed secondary amide with another carbonyl group.

On the other hand, as we previously reported [8], n-octylamine can also produce a silica nitridation [54,55]. In a previous paper, Han et al. [55] investigated interactions between methylamine and zeolites. They concluded that the strong hydrogen bonding interaction results in the H atom of the amine group attacking the Si–O framework to form a Si–O...H...N bond, which leads to the formation of Si–N bonds in the zeolites. We think that a similar silica nitridation process could occur in the UCA products. Finally, the band at 1342 cm^−1^ could be assigned to the C–N vibration of n-octylamine [8,56]. It confirms the presence of residual n-octylamine in the mix.

All the previously described findings allow it to be concluded that the presence of n-octylamine in the UCA product can promote its suitable adhesion to the Acinipo limestone.

In order to confirm the long-term effectiveness of the products after three years exposure to archaeological site conditions, the spectra before and after exposure of the limestone samples were obtained (Supplementary Information, Figure S7). As a relevant feature, the silica bands, which are located at 1080 and 800 cm^−1^ [7,48], are more intense in the stone treated with the UCA product, and they remained after three years of exposure. It confirms the long-term effectiveness of the UCA product.

#### 3.2.2. Effectiveness of the Products on the Limestone

**Figure 10:**
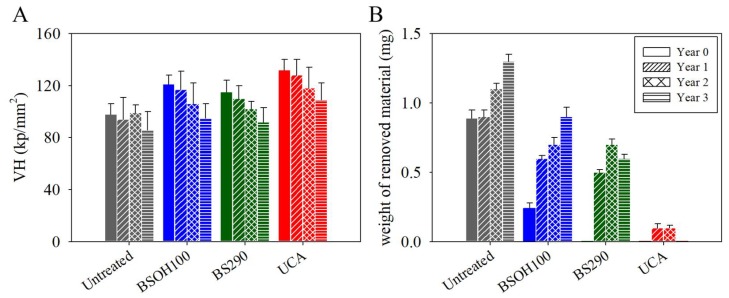
(**A**) Vickers hardness (VH) values and (**B**) weight of removed material of the treated and untreated stones, before and after three years of exposure to the environmental conditions of the Acinipo archaeological site.

The consolidant effectiveness of the products on the stone surface was evaluated by the Vickers hardness test. The hardness values (VH) are shown in Figure 10. The results indicate that all products increase the resistance of the limestone surface when compared to the untreated samples. In particular, the surface that was treated with the UCA product shows the highest increase in hardness (36%), whereas BSOH100 and BS290 produce increases of 23% and 17%, respectively. This highest increase for UCA can be associated with the formation of a continuous and homogeneous coating [6,7], while the commercial products produced discontinuous coatings, as observed by SEM (Figure 6). All of the treatments present a gradual reduction in the Vickers hardness with time of exposure. The commercial products BSOH100 and BS290 show the higher reduction, with values that were approaching the values of the untreated stone after three years of exposure, whereas the UCA product maintains the highest values of hardness. These results newly highlight the low durability of the commercial products.

The material cohesion and adhesion of the coatings were evaluated by a peeling test. Figure S8 shows photographs of the adhesive tapes after being applied to the surface of the limestone samples, before and after three years of exposure to archaeological site conditions. Before archaeological site exposure, the untreated limestone and the samples that were treated with BSOH100 present a material loss, whereas after three years of exposure, all the surfaces, excepting that treated with UCA, show material loss due to the alteration of the surface during exposure to environmental conditions. The Figure 10 presents the corresponding weight removed from the limestone surfaces. All the treated stones show higher resistance than their untreated counterpart. The untreated sample, and the samples that were treated with the BSOH100 and BS290 products, present an increase in the weight of the material removed with increasing time of exposure, whereas the material removed from the surface treated with the UCA product was practically negligible after three years of exposure to archaeological site conditions. These results newly confirm the long-term effectiveness of UCA products under Acinipo archaeological site conditions.

**Figure 11:**
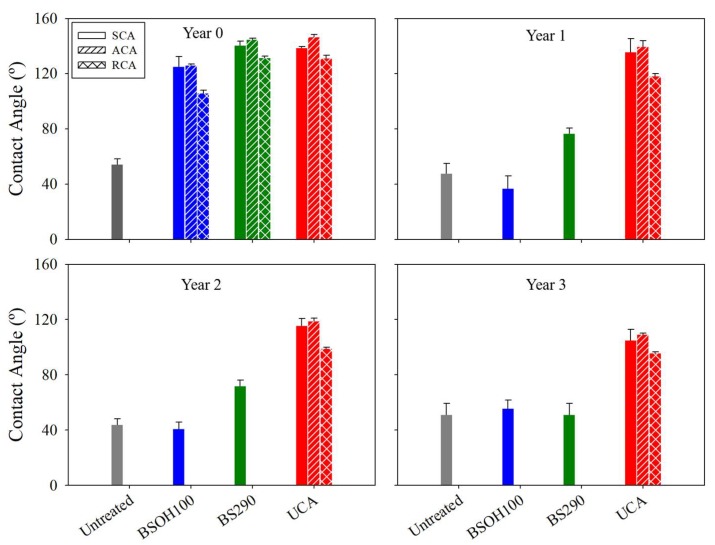
Static contact angle (CA) (SCA) values and their corresponding advancing (ACA) and receding CA (RCA) values, for the treated and untreated stones, before and after three years of exposure to the environmental conditions of the Acinipo archaeological site.

The durability of the hydrophobic properties of all the investigated products was evaluated by a study of the static and dynamic contact angles. The contact angle (CA) values obtained on the treated and untreated limestone surfaces are shown in Figure 11. Figure S9 in supplementary information shows images of water droplets deposited on Acinipo limestone samples, before and after three years of exposure to Acinipo conditions. The untreated limestone presents a static CA of around 50°. The dynamic CA could not be evaluated due to the hydrophilic nature of the stone. Before exposure, all of the treated limestones show receding angles higher than 90° (106, 132, and 131° for BSOH100, BS290 and UCA, respectively), essential requirements for a product to be able to prevent the penetration of water into the stone [57]. The BSOH100 product, without stable organic groups in its composition, confers hydrophobic properties to the stone due to the slow hydrolysis process of ethoxy groups, as discussed in a previous paper [9]. The hysteresis values, the difference between advancing and receding CA, which characterize repellence [37], were significantly lower for the limestones that were treated with the two hydrophobic products evaluated.

After the limestone exposure to the archaeological site conditions, a progressive reduction in static CA over time was observed. After three years of exposure, the static CA for the limestones that were treated with the two commercial products practically presented the same value as that corresponding to the untreated stone. The dynamic CA could not be evaluated in any case, confirming the loss of repellence properties. The surface that was treated with the UCA product maintained the value of the static and dynamic CA after one year of exposure (Figure 11). A progressive reduction in CA is observed for the following years, showing a static CA of around 110° and a hysteresis of 15° after three years. These results allow it to be concluded that UCA was the only product maintaining hydrophobic properties after three years of exposure. The reduction in CA can be related to the coatings removal and the change in surface roughness, observed after limestone exposure under site conditions [14].

**Figure 12:**
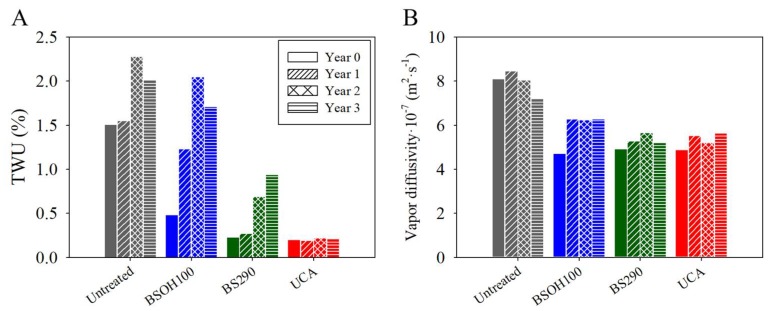
(**A**) Total water uptake (TWU) and (**B**) water vapour permeability values of treated stone and their untreated counterpart, before and after three years of exposure to the environmental conditions of the Acinipo archaeological site.

In order to confirm the results that were obtained by the CA evaluation, a water absorption test by capillarity was carried out (see Figure 12). Before the archaeological site exposure, the untreated samples showed the highest total water uptake (TWU) values, followed by the limestone samples treated with the BSOH100 product. These results confirm that the commercial BSOH100 had lost its hydrophobic capacity in contact with water during the capillary absorption test due to the complete hydrolysis of the ethoxy groups [14]. In the case of the samples that were treated with the UCA and BS290 products, with hydrophobic properties, low water absorption, close to zero, was observed.

After three years of exposure to Acinipo conditions, the untreated samples and their counterpart treated with BSOH100 showed the same trend: a gradual increase, with slight fluctuations, in the water absorption. It could be associated to a significant loss of the coatings over the exposure time (see Figure 6). In the case of the samples that were treated with the UCA product, the water absorption values were maintained at around 0.2%, without visible changes during the three years of exposure. Moreover, a direct relationship between the CA and TWU values was observed. The surfaces with receding CA values lower than 90°, observed for all of the samples except for those that were treated with UCA, showed significant water absorption by capillarity.

#### 3.2.3. Negative Effects Induced by the Products on the Limestone

The changes in the water vapor permeability and in the color of the Acinipo Limestone induced by the products were evaluated. The values of water vapor diffusivity are given in Figure 12. All of the products promoted a reduction of the vapor diffusivity coefficient of below 50%. It highlights that breathability of the stone was only slightly reduced, these results being acceptable for use in cultural heritage preservation [27,58]. After three years of exposure to Acinipo conditions, all of the products maintained the reduction in diffusivity below the threshold of 50%.

**Figure 13:**
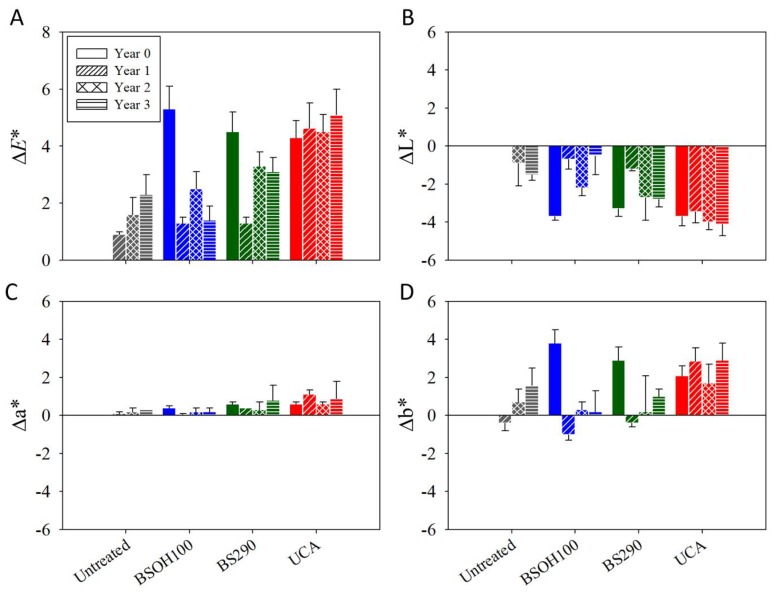
Colour data for the treated limestone and their untreated counterpart, before and after three years of exposure to the environmental conditions of the Acinipo archaeological site. (**A**) Total colour difference (ΔE*); (**B**) Black-white (ΔL*); (**C**) Green-red (Δa*); (**D**) Yellow-blue (Δb*).

In addition, the color variations produced by the different products were evaluated, by using the color differences that were obtained in coordinates (ΔL *, Δa *, Δb *) and the ΔE* (total colour difference). The values that were obtained are shown in Figure 13. Before exposure to the archaeological site conditions, the samples treated with the product BSOH100 showed total colour difference values of around 5, and the products BS290 and UCA values were below 5, which is the maximum color change acceptable for cultural heritage materials [59]. The color variations are due to darkening (negative values of ΔL*) and yellowing (positive values of Δb *) of the samples after being treated.

After the exposure of samples to Acinipo conditions, the samples that were treated with the commercial products BSOH100 and BS290 presented a progressive reduction in the values of ΔE*. This change is probably due to the partial or total loss of the coating in the samples treated with the commercial products, after exposure to the outdoor conditions, as previously described. Therefore, these samples tend to recover their initial color. In the case of the samples that were treated with the UCA product, the total color difference value recorded before Acinipo exposure remained practically unaltered, confirming the durability of this product after exposure to the outdoor conditions for three years.

#### 3.2.4. Evaluation of the Depth of the Products Penetration

**Figure 14:**
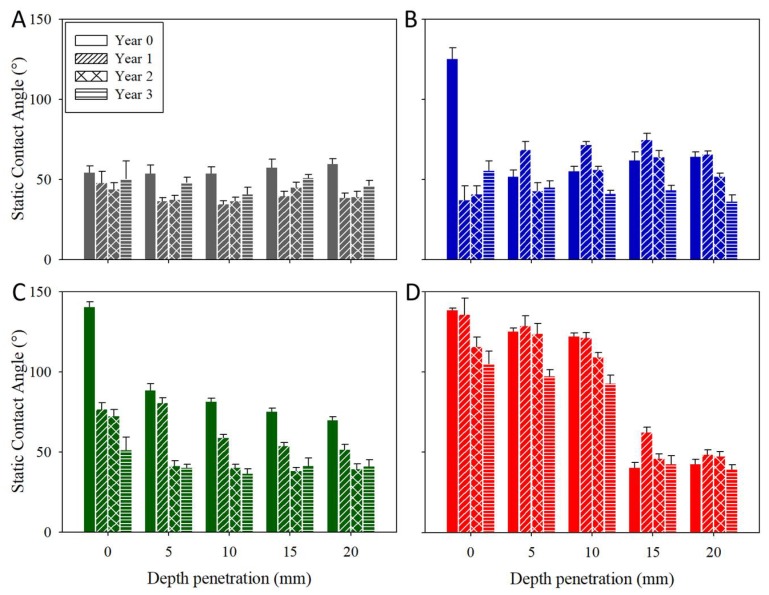
Static CA values on the cross-sections of the untreated and the treated limestone, before and after three years of exposure to the environmental conditions of the Acinipo archaeological site. (**A**) Untreated; (**B**) BSOH100; (**C**) BS290; (**D**) UCA.

The depth of penetration was determined based on the hydrophobic effect of the products. The penetration of conservation products is important to achieve a durable treatment against the atmospheric agents [60]. In this regard, the static CA values of water droplets deposited at different depths on the cross-section of the samples were determined. The results that were obtained are shown in Figure 14. Before the archaeological site exposure, the samples that were treated with the products under study show CA values in the surface ranging from 125 to 140°, whereas the untreated surface shows CA values of around 50°. However, for all of the depths evaluated, the samples treated with the two commercial products exhibit CA values of around 50°. In the case of the limestone that was treated with UCA, the static CA is maintained above 90° up to 10 mm of depth. These results show that the UCA product is able to adhere to and penetrate into the porous structure of the stone, whereas the other products form a surface coating. After the Acinipo exposure, the same trend is observed with slight reductions in the CA values.

**Figure 15:**
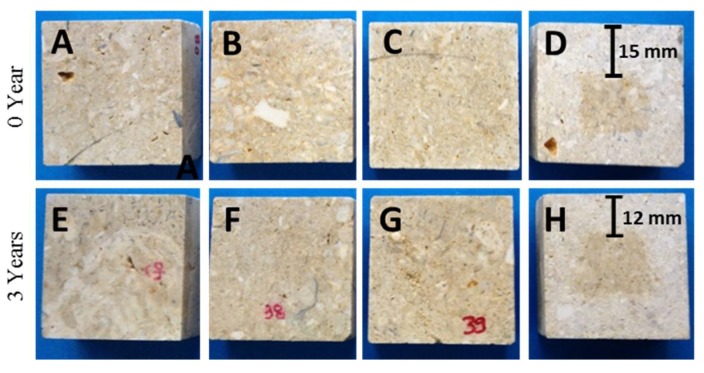
Photographs of cross-sections of the limestone samples, before and after exposure to the environmental conditions of the Acinipo archaeological site: (**A**,**E**) untreated; (**B**,**F**) BSOH100; (**C**,**G**) BS290; and (**D**,**H**) UCA.

In order to confirm the results that were obtained by CA measurement, Figure 15 shows photographs of the cross-sections of treated and untreated samples after immersion in water. In the case of the untreated samples and their counterparts treated with the commercial products, all of the surfaces are completely wet, confirming the low penetration of the products, as previously observed for BS290 applied by spraying on roof tiles [14]. The samples treated with the UCA product, show a dry zone of up to 15 mm of depth, before exposure to outdoor conditions, which confirms the existence of product up to that depth. After three years of exposure, the penetration depth of UCA remains unchanged. These results confirm that the BSOH100 and BS290 products do not penetrate into the pore structure of the stone, whereas the UCA product is able to penetrate into the pore structure of the stone, increasing the durability of the treatment against the influence of outdoor conditions.

## 4. Conclusions

This study represents an investigation about the long-term effectiveness of a novel stone conservation nanomaterial by exposure to real conditions. The following conclusions has been obtained:

- The environmental study carried out in the Acinipo Archaeological site highlights that the limestone decay was caused by salts, deposition of other pollutants, and the growth of microorganisms, with water being the main vehicle for the decay agents. Thus, effectiveness of an innovative alkoxysilane-based product with hydrophobic and consolidant properties, preventing water ingress, was tested on limestone samples that were extracted from the Acinipo Roman Theatre.
- The evaluated commercial products showed a poor compatibility with the carbonate stone under study, producing discontinuous aggregates on its surface. In addition, a low penetration and an absence of long-lasting performance were observed for these commercial products.
- In the case of the innovative nanomaterial under study, an adequate compatibility and adherence to the limestone, producing a long-term effective, homogeneous, and continuous coating with a depth of penetration of up to 10 mm, were observed. This compatibility was explained in terms of the presence of effective interactions between the n-octylamine integrated in the product and the carbonate substrate.

Negative effects, including significant changes in color and reduction in water vapor diffusivity, were not induced by the products under study.

## 5. Patents

The product under study was previously patented (Spanish Patent N° ES2423356. Priority Date: 16 February 2012).

## Supplementary Materials

The following are available online at http://www.mdpi.com/1996-1944/11/5/694/s1, Figure S1: (A) Monthly temperature (with minimum and maximum temperatures); (B) monthly rainfall (averaged from 2011–2012), Figure S2: (A) Optical microscopy photographs and (B) EDX spectrum of the Acinipo limestone, Figure S3: SEM images of the microorganisms on the limestone surface, after 3 years of exposure to outdoor conditions: (A) Untreated; (B) BSOH100; (C) BS290 (D) UCA, Figure S4: (A) Images of limestone samples in PDA culture medium and (B) Images obtained by OM of the microorganisms, Figure S5: Pore size distributions of the treated and untreated stones, before and after 3 years of exposure to outdoor conditions, Figure S6: FTIR spectra of the Acinipo limestones without and with catalyst (n-octylamine), Figure S7: FTIR spectra of the treated and untreated limestone, before and after 3 years of exposure to outdoor conditions, Figure S8: Adhesive tape applied to the surface of untreated and tared limestones, (A) before and (B) after 3 years of exposure to outdoor conditions, Figure S9: Images of droplets water deposited on limestone surface, before and after exposure. (A,E) Untreated; (B,F) BSOH100; (C,G) BS290; (D,H) UCA.
