# Supplementary material for: Long-Term Effectiveness, under a Mountain Environment, of a Novel Conservation Nanomaterial Applied on Limestone from a Roman Archaeological Site
Source: Materials (Basel). 2018 Apr 28;11(5):694. doi: 10.3390/ma11050694 (PMC5978071; doi:10.3390/ma11050694)
Supplement: Supplementary file 1 [file materials-11-00694-s001.docx]

**Supplementary information**

Long-term effectiveness, under a mountain environment, of a novel conservation nanomaterial applied on limestone from a Roman archaeological site

Farid Elhaddad, Luis A.M., Maria J. Mosquera*

TEP-243 Nanomaterials Group. Departamento de Química-Física, Facultad de Ciencias, Campus Universitario Río San Pedro, Universidad de Cádiz, 11510 Puerto Real, Cádiz, Spain.

*Corresponding author e-mail: mariajesus.mosquera@uca.es.

Phone: (34) 956016490 (34)95601631.


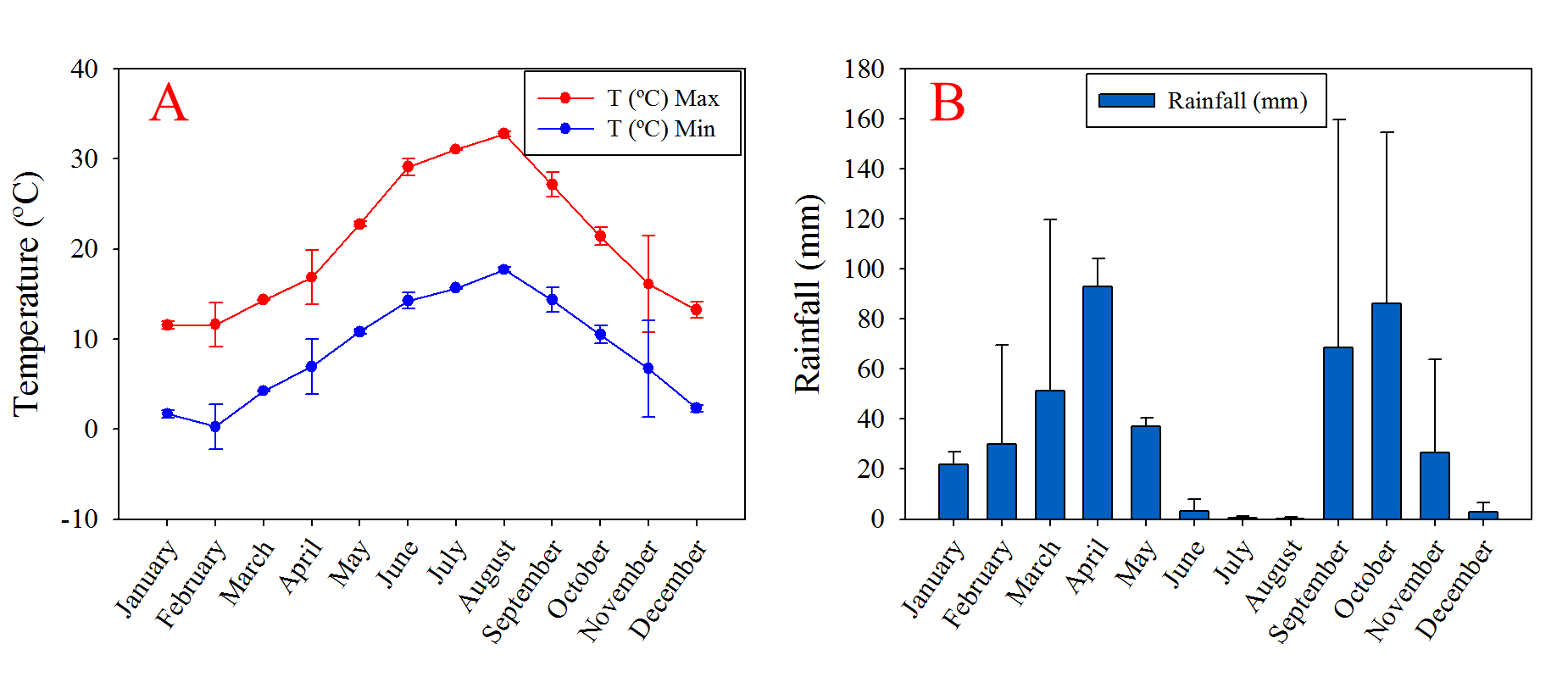


Fig.S1. (A). Monthly temperature (with minimum and maximum temperatures). (B) monthly rainfall (averaged from 2011-2012).

**
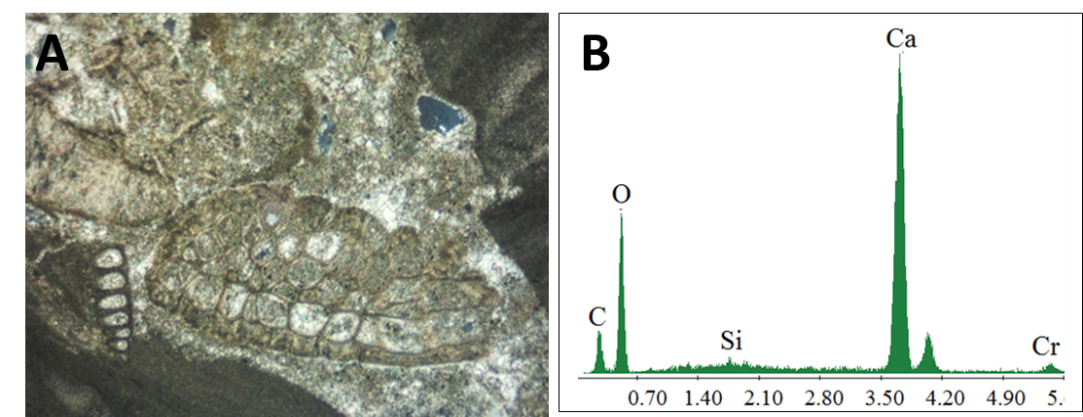
**

Fig. S2. (A) Optical microscopy photographs and (B) EDX spectrum of the Acinipo limestone.


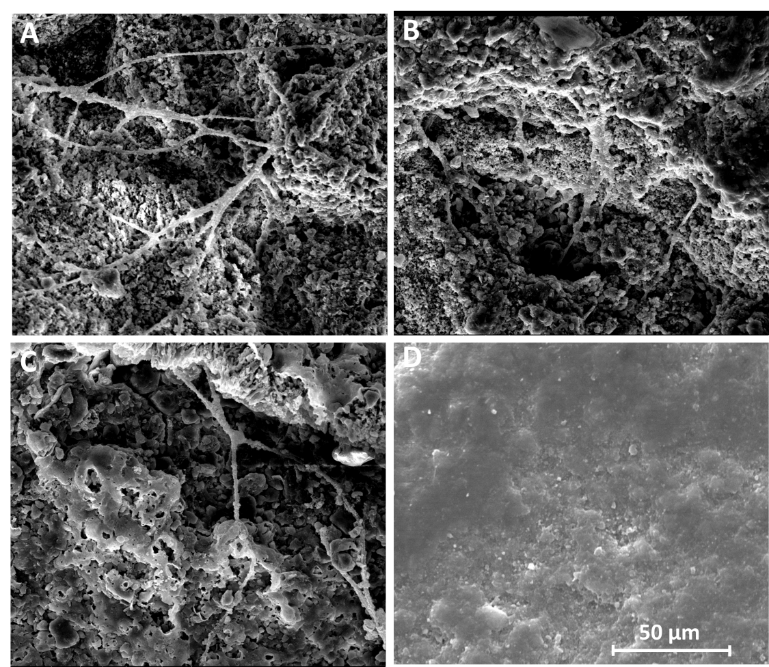


Fig. S3. SEM images of the microorganisms on the limestone surface, after 3 years of exposure to outdoor conditions: (A) Untreated, (B) BSOH100, (C), BS290 (D) UCA.

**
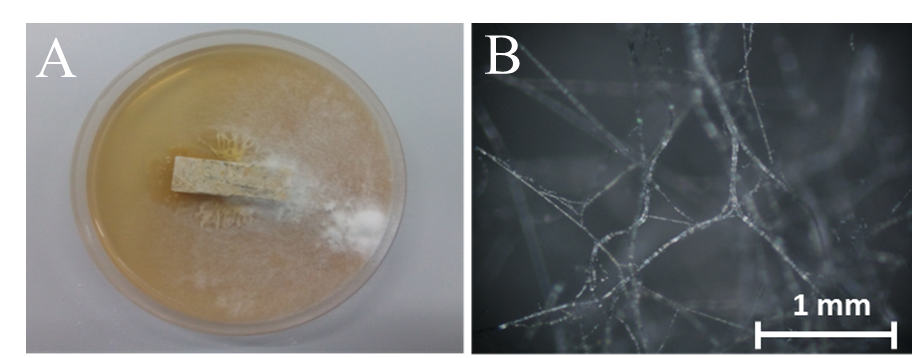
**

Fig. S4. (A) Images of limestone samples in PDA culture medium and (B) Images obtained by OM of the microorganisms.

Table S1. Uptake and dry-matter values of the products applied on the stone samples.

| Product | Uptake, (U, %w/w) | Dry-matter (D, % w/w) | D/U ratio |
| --- | --- | --- | --- |
| BS290 | 1.28 ± 0.27 | 0.58 ± 0.13 | 45.31 |
| BSOH100 | 0.80 ± 0.12 | 0.13 ± 0.05 | 16.25 |
| UCA | 1.34 ± 0.11 | 0.93 ± 0.12 | 69.40 |


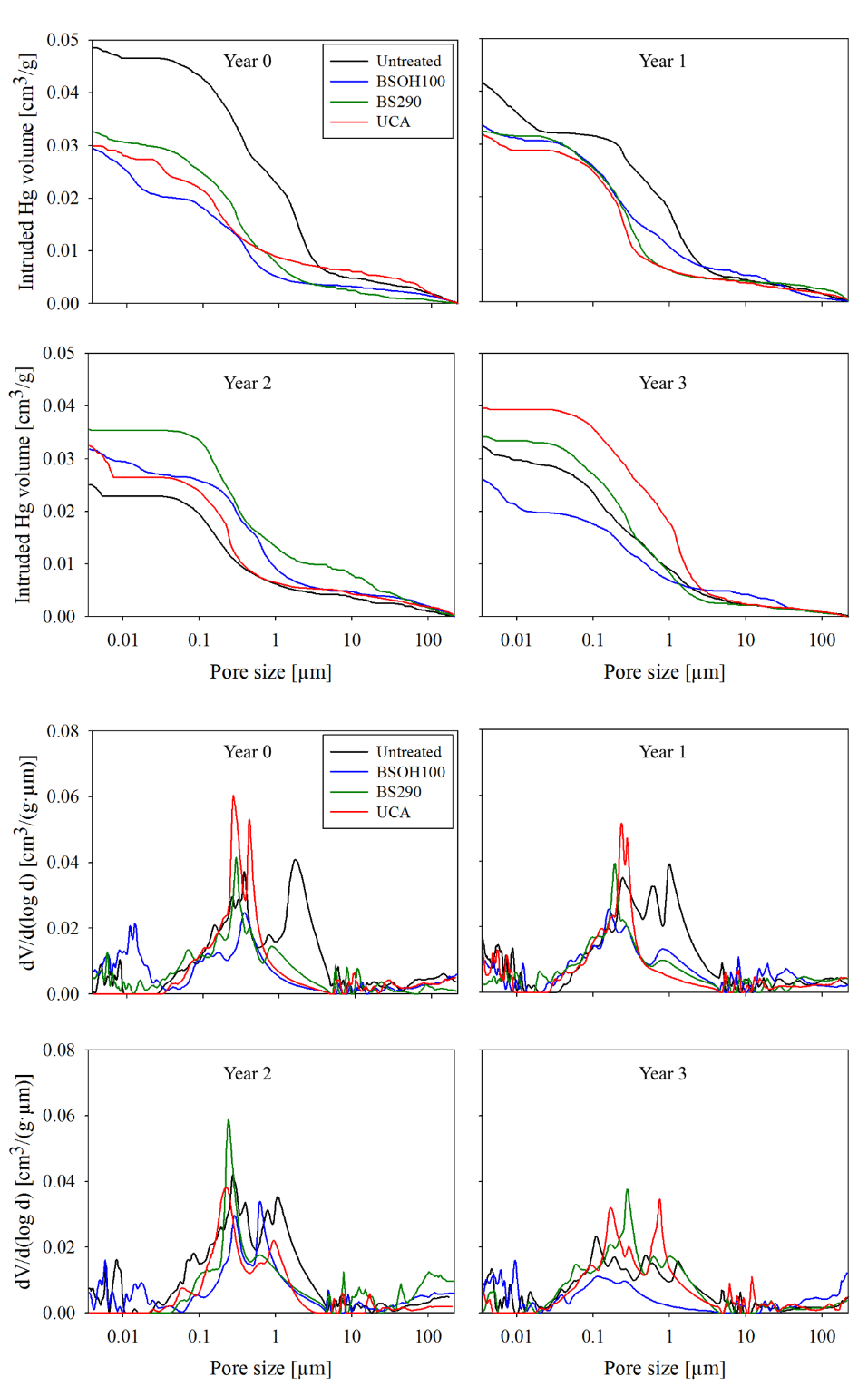


Fig. S5. Cumulative and differential pore size distribution of the treated and untreated stones, before and after 3 years of exposure to outdoor conditions.

Table S2. Relative intensity of the main siloxane band (1080 cm^-1^) observed in the FTIR spectra of Figure 9. The relative intensity is calculated respect to the intensity of the main CO_3_^2-^ band at 1400 cm^-1^.

| Product | 1080 cm^-1^ (Si-O) |
| --- | --- |
| Untreated | 0.76 |
| BS290 | 0.93 |
| BSOH100 | 0.78 |
| UCA | 1.08 |


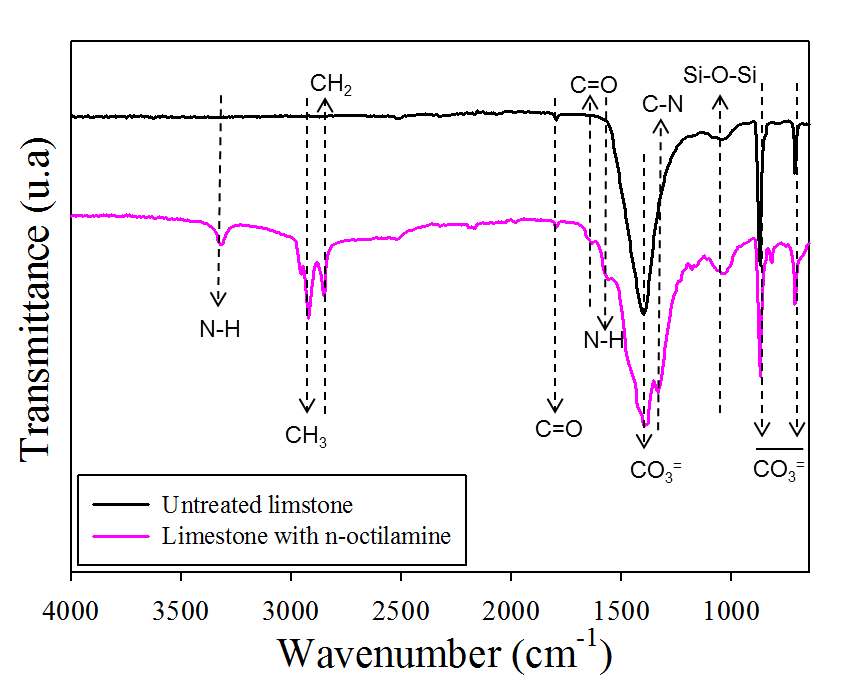


Fig. S6. FTIR spectra of the Acinipo limestones without and with catalyst (n-octylamine).


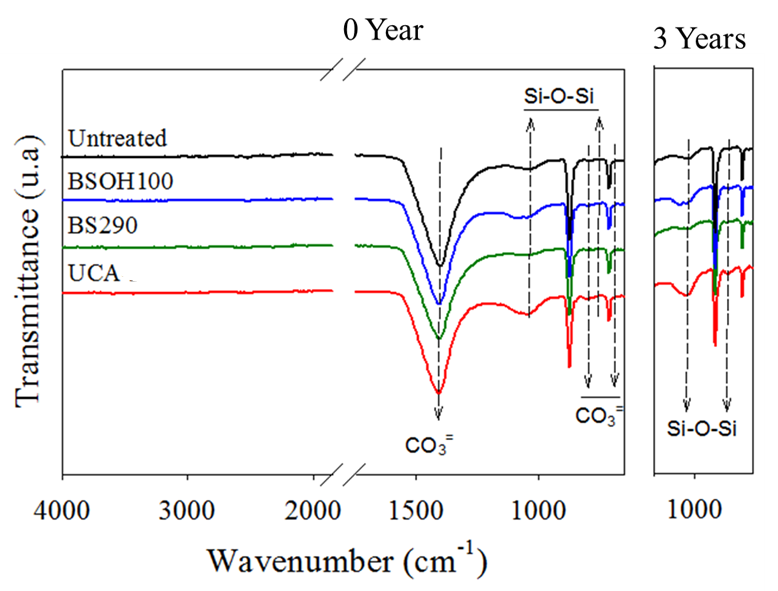


Fig. S7. FTIR spectra of the treated and untreated limestone, before and after 3 years of exposure to outdoor conditions.


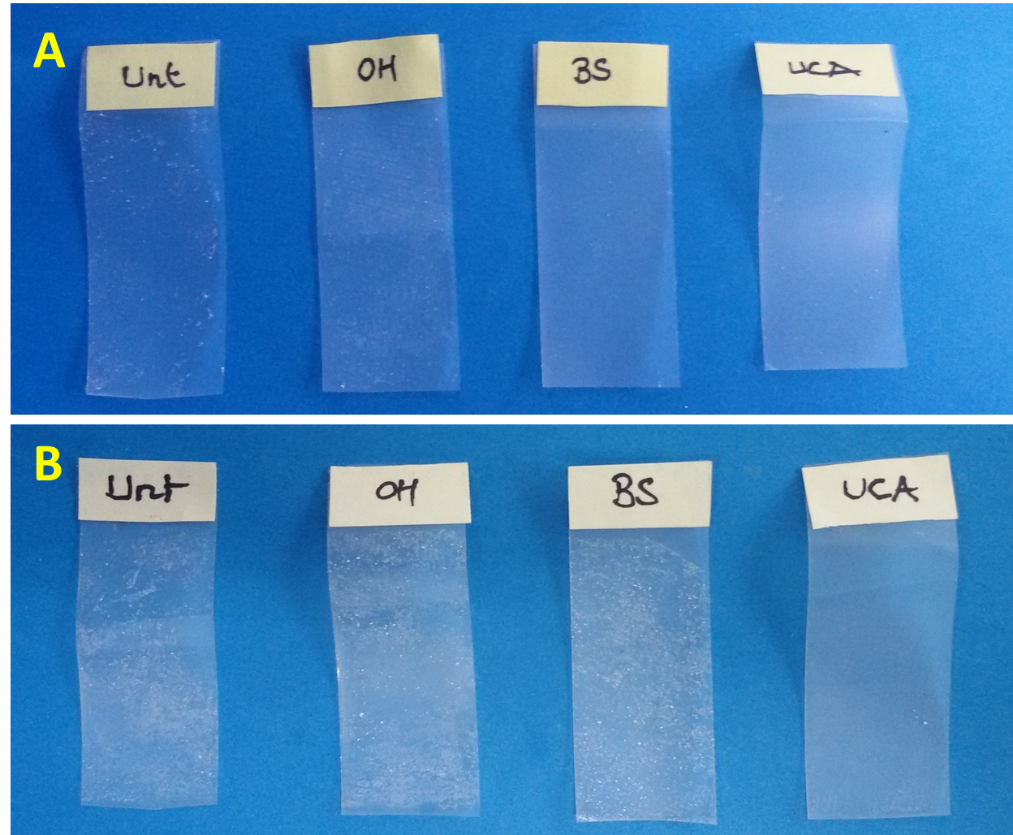


Fig. S8. Adhesive tape applied to the surface of untreated and tared limestones, (A) before and (B) Afetr 3 years of exposure to outdoor conditions.


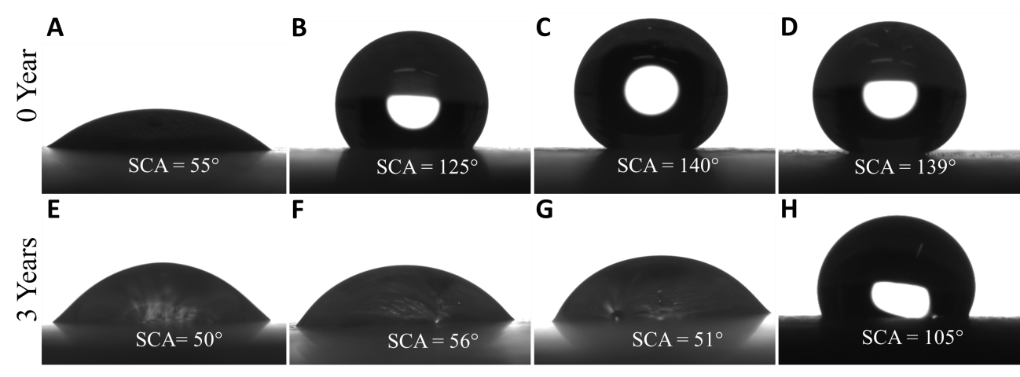


Fig. S9. Images of droplets water deposited on limestone surface, before and after exposure. (A, E) Untreated, (B, F) BSOH100, (C, G) BS290, (D, H) UCA.
